# Transplantation of a sheet of human corneal endothelial cell in a rabbit model

**Published:** 2008-01-03

**Authors:** Kouichiro Hitani, Seiichi Yokoo, Norihiko Honda, Tomohiko Usui, Satoru Yamagami, Shiro Amano

**Affiliations:** 1 Department of Ophthalmology, University of Tokyo Graduate School of Medicine; 2 Department of Ophthalmology, Toho University Graduate School of Medicine; 3 Department of Corneal Tissue Regeneration, University of Tokyo Graduate School of Medicine, Tokyo, Japan

## Abstract

**Purpose:**

To develop a novel method for constructing a sheet of human corneal endothelial cells (HCECs) and examine the properties of the HCEC sheet.

**Methods:**

HCECs were cultured on a cell culture insert for a week; ethylenediamine tetraacetic acid was applied from the bottom of the cell culture insert to attenuate the attachment of HCECs. The sheet of HCECs was constructed by bluntly detaching the cell sheet with a spatula. HCEC cell sheets were placed on the posterior surface of excised rabbit corneal buttons and transplanted onto the corneal beds of donor rabbits. In two eyes from the HCEC sheet group, cultured HCECs were labeled with PKH26 to observe the localization of HCECs after transplantation.

**Results:**

Cultured HCECs could be bluntly detached *en bloc* from the bottom of a culture insert. Immunostaining for ZO-1, Na^+^, K^+^-ATPase, laminin, fibronectin, and type IV collagen was positive in the cell sheet. The average cell density in a HCEC sheet was 2,425 cells/mm^2^. After HCEC sheet transplantation, corneal edema decreased much earlier in the HCEC group than in the control group. In the HCEC sheet group, the monolayer of continuous cells attached to the posterior surface of the transplanted rabbit cornea and the posterior surface of transplanted cornea was covered with PKH26-labeled cells. The average endothelial cell density in the HCEC sheet group seven days postoperatively was 2,244 cells/mm^2^.

**Conclusions:**

This technique for producing an HCEC sheet might be useful in regenerative medicine for the cornea and reconstruction of the corneal endothelium.

## Introduction

Corneal endothelial cells reside on the innermost layer of the cornea and contribute to maintenance of the corneal transparency by their pump and barrier function. When corneal endothelium decomposes due to various causes, such as intraocular surgeries and Fuchs dystrophy, several kinds of surgical procedures are indicated, including penetrating keratoplasty, deep lamellar endothelial keratoplasty [1,2], Descemet's stripping, and endothelial keratoplasty [3,4]. However, all of these surgeries need donor corneas, which are in grave shortage world wide.

Although human corneal endothelial cells (HCECs) have limited proliferative capacity in vivo [5,6], they can be propagated by cell culture [7-11]. Cultured human or animal corneal endothelial cells were seeded onto animal corneal stroma and were successfully transplanted in animal models [12-22]. Several studies reported transplantation of corneal endothelial cells cultured on carriers such as a gelatin membrane [23], amniotic membrane [24], and collagen sheet [25] in animal models. However, usage of carriers might affect the transparency of the cornea and facilitate contamination by pathogens. Thus, transplantation of only corneal endothelial cells is ideal. Recently, others have reported a method to produce a sheet composed of corneal endothelial cells using temperature sensitive culture dishes [26-28]. However, their method needs special culture dishes coated with poly(N-isopropylacrylamide), which had cytotoxic effects [29]. We developed a novel method for constructing a sheet of corneal endothelial cells and examined the properties and in vivo function of the sheet.

## Methods

### Media and culture conditions for human corneal endothelial cells

Primary cultures of HCECs were performed as described previously [11]. Briefly, primary cultures of HCECs were established from the remainder of a donor cornea after penetrating keratoplasty. The donor corneas were obtained from the Rocky Mountain Lion's Eye Bank. The donor for this study was 10 years of age. All primary cultures and serial passaging of HCECs were performed in growth media consisting of low glucose Dulbecco's modified Eagle medium (DMEM) supplemented with 15% fetal bovine serum (FBS), 2.5 mg/l fungizone (Gibco BRL, Grand Island, NY), 2.5 mg/l doxycycline, and 2 ng/ml basic fibroblast growth factor (bFGF; Sigma, St. Louis, MO). Small explants from the endothelial layer, including the Descemet's membrane, were removed with sterile surgical forceps. About 200 explants were made per cornea and were placed endothelial cell-side down onto four 35 mm culture dishes coated with bovine extracellular matrix. Bovine extracellular matrix was produced by culturing bovine corneal endothelial cells on culture dishes as previously described [11]. The dishes were carefully placed in the incubator. After three days, the media was exchanged and was replaced every two days thereafter. When a sufficient density of proliferating cells had been reached, HCECs were passaged at ratios ranging from 1:1 to 1:4. All subsequent passaging was performed using the same method, but at a ratio of 1:16. Cultured cells from the fifth passage were used in this study.

### Human corneal endothelial cell sheet production using culture inserts

**Figure 1:**
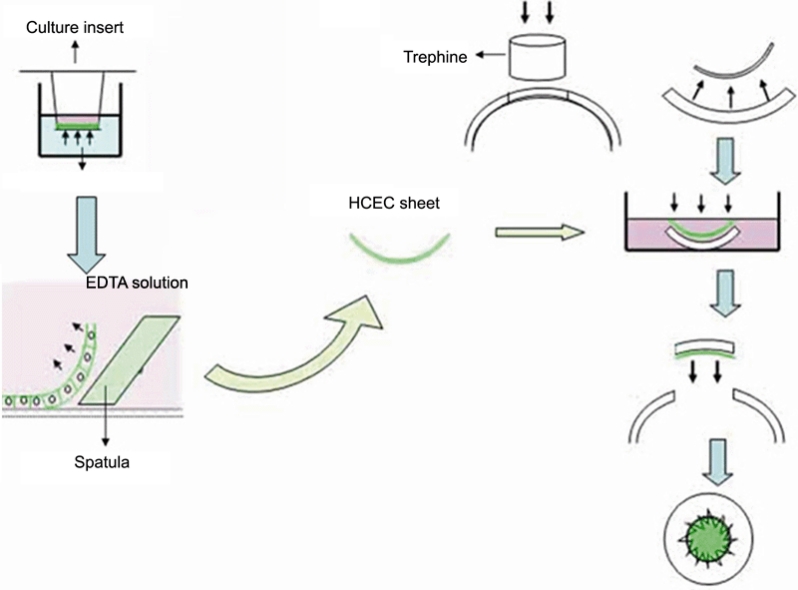
Schema illustrating preparation and surgical procedure of human corneal endothelial cell sheet transplantation. A cell sheet of HCECs was constructed by bluntly detaching the cell sheet from the bottom of a culture insert with a spatula after the application of EDTA. The HCEC cell sheet was attached to a posterior surface of an excised rabbit corneal button and transplanted to the corneal bed in the donor rabbit.

To produce HCEC sheets, cell culture plates with 12 wells and culture inserts (BD Biosciences, San Jose, CA) were used. The culture insert had a diameter of 10.5 mm and the membrane on the culture insert had a pore size of 0.4 μM. A HCEC cell suspension of 4.0x10^6^ cells in 600 μl culture media (low-glucose DMEM containing 6% dextran) was transferred onto the membrane of the culture inserts. These culture inserts were placed in the wells of 12-well plates and centrifuged at 1,000 rpm (176 g) for 5 min to enhance cell attachment to the membrane. The cells were incubated at 37 °C with 5% CO_2_ and the media was exchanged every two days. After one week, the culture insert was placed on a sterile dish and the bottom of the membrane in the culture insert was washed three times with PBS (-). Then, ethylenediamine tetraacetic acid (EDTA) was applied only from the bottom of the cell culture insert for 1 h to attenuate attachment of HCECs to the bottom of the culture dishes. Then, the membrane of the culture insert with HCEC was cut out and HCECs were bluntly detached *en bloc* from the underlying membrane of the culture insert with a spatula, thus yielding a HCEC sheet (Figure 1).

### Histologic analysis

Three HCEC sheets were fixed with 10% formalin and processed for light microscopic examination. The specimens were stained with hematoxylin and eosin. Three HCEC sheets for electron microscopic observation were immersed for 10 min in a fixative consisting of 2.5% glutaraldehyde in 0.1 M PBS at pH 7.4, segmented in the fixative, and immersed in the fixative for 12 h. They were osmicated in 1% OsO4 in the same buffer for 1 h, and dehydrated in ethanol. The tissues were embedded in Epon 812. Tissue sections (1 μm thick) were cut using glass knives, stained with toluidine blue, and observed using a light microscope. Thin sections were stained with lead citrate and uranyl acetate, and examined with a transmission electron microscope (Model 1200EX, Nihon Denshi, Tokyo, Japan).

### Immunohistochemistry

Immunohistochemical analysis was performed on three HCEC sheets. Each HCEC sheet was placed onto a slide glass for flat mount observation and was fixed with 99.8% methanol for 10 min. After rinsing with PBS, the sheet was incubated for 30 min with 3% BSA (Sigma-Aldrich, St. Louis, MO) in PBS containing 0.3% Triton X-100 (Rohm & Haas, Philadelphia, PA) to prevent non-specific staining. After two 5-min rinses with PBS, the sheet was incubated for 2 h at room temperature with rabbit anti-ZO-1 antibody (1:150; ZYMED Laboratories, South San Francisco, CA) and mouse anti-Na^+^, K^+^-ATPase α-1 antibody (1:50; Upstate Biotech, Lake Placid, NY). Mouse IgG or rabbit IgG were used as controls. After three washes with PBS(-), the sheets were incubated at room temperature for 1 h with the appropriate secondary antibody, fluorescence-labeled goat anti-mouse IgG (Alexa Fluor 488, 1:200; Molecular Probes, Eugene, OR) or anti-rabbit IgG (Alexa Fluor 488, 1:200; Molecular Probes). After another three washes, the sheets were mounted with anti-fading mounting medium containing propidium iodine (Vectashield; Vector Laboratories Burlingame, CA) that simultaneously counterstains DNA in the nuclei. Images were obtained under a fluorescence microscope (BX50; Olympus, Tokyo, Japan) and were saved to a personal computer. The cell number in a 0.1 mm by 0.1 mm square of a ZO-1 stained sample was counted at four different sites in three HCEC sheets and the average cell density was calculated.

To examine the expression of extracellular matrix, frozen 6 μM-thick sections of HCEC sheets were prepared using OCT compound (Tissue-Tek; Sakura Fine Tek, Torrance, CA). The immunohistochemical procedure was as mentioned above. The primary antibodies included mouse anti-fibronectin antibody (1:150; BD Biosciences, Billerica, MA), rabbit anti-laminin antibody (1:50; Sigma-Aldrich), and mouse anti-human collagen type IV antibody (1:50; Chemicon International).

### Transplantation of human corneal endothelial cell sheet into rabbits

All animals were treated in accordance with the ARVO Statement on the Use of Animals in Ophthalmic and Vision Research. All rabbits were obtained from Saitama Experimental Animals Inc., Japan (Saitama, Japan). Twelve New Zealand white rabbits weighing 2.0–2.4 kg, were anesthetized with intramuscular ketamine hydrochloride (60 mg/kg; Sankyo, Tokyo, Japan) and xylazine (10 mg/kg; Bayer, Leverkusen, Germany). After disinfection and sterile draping of the operation site, the central cornea was trephined using a 6.0-mm diameter Hessburg-Barron trephine (Katena products Inc., Denville, NJ). Rabbit corneal endothelium and Descemet's membrane on the corneal button were totally removed using forceps. A HCEC sheet was placed onto the denuded posterior surface of the rabbit corneal button and left to attach for 15 min. The HCEC sheet on the rabbit corneal stroma was placed in a six-well culture plate and centrifuged at 1,000 rpm (176 g) for 3 min to enhance the attachment of the sheet onto the rabbit corneal stroma. The rabbit corneal stroma with the HCEC sheet was sutured to graft bed of the same rabbit with a combination of four interrupted sutures and a continuous suture using 10–0 nylon (Mani, Tochigi, Japan). Ofloxacine ophthalmic ointment (Santen, Osaka, Japan) was instilled immediately after the operation (Figure 1). In addition, ofloxacin ophthalmic ointment and 0.1% phosphate betamethasone were instilled once a day during the week following the surgery.

Rabbits were classified into two groups: HCEC sheet group (rabbits with transplantation of a HCEC sheet) and control group (rabbits with peeling of Descemet's membrane and corneal endothelial cells without transplantation of a HCEC sheet). Each group consisted of six right eyes of six rabbits.

In two eyes of the HCEC sheet group, cultured HCECs were labeled with PKH26 (Sigma-Aldrich, St. Louis, MO) to observe localization of the HCECs after transplantation. The trypsinized 4.0x10^5^ HCECs were incubated in 1 ml of PKH26 solution (4x10^−6^ M) for 5 min at room temperature; media containing 15% FBS was then added to stop the reaction. After labeling, the HCECs were washed twice with PBS and re-suspended in 300 μl low glucose DMEM.

### Anterior segment observations

Each operated eye was observed with a slit-lamp microscope (models BH2-RFL-T3 and BX50; Olympus, Tokyo, Japan) and photographed for seven days after the surgery. Central corneal thickness was measured with an ultrasound pachymeter (SP-2000; Tomey, Nagoya, Japan) during the week after the surgery; an average of three readings was taken. The differences in corneal thickness between the HCEC sheet group and the control group at each time point were evaluated using the unpaired *t*-test. The p-value for statistical significance in this evaluation was set to p=0.007 following Bonferroni correction for multiple comparisons, since the corneal thickness was compared at seven different postoperative time points.

### Histologic examination and localization of human corneal endothelial cells after transplantation

Under deep anesthesia, rabbits were sacrificed with an overdose intravenous injection of pentobarbital sodium (Dainippon Pharmaceutical, Osaka, Japan); corneas were excised from each rabbit at one week after transplantation. Each cornea was cut in two. After staining with 0.2% alizarin red for 1 min, the morphologies of the HCECs were evaluated on one side of the four divided corneas. All plates were examined under a light microscope (model BX-50; Olympus) and images were saved to a personal computer. The cell number in a 0.1 mm by 0.1 mm square was counted at four different sites in two HCEC sheets on the rabbit corneal stroma. The other side of the four divided corneas was fixed in 10% formalin (Wako Pure Chemicals, Osaka, Japan). The samples were embedded in OCT compound (Tissue-Tek®; Miles Laboratories, Naperille, IL) at −20 °C. Frozen OCT-embedded sections were cut at 8 μm thickness, placed on silane-coated microscope slides (Muto, Tokyo, Japan), stained with hematoxylin and eosin (HE) and observed with light microscopy. The other two corneas of the HCEC sheet group, in which HCEC was labeled with PKH26, were observed to examine fluorescence as a whole mount sample under a fluorescence microscope (model BH2-RFL-T3 and BX50; Olympus) with an excitation wavelength of 420 nm and emission wavelength of 480 nm. Thereafter, these samples were immersed in a fixative consisting of 4% paraformaldehyde (Wako) in 0.1 M PBS at pH 7.4, and embedded in OCT compound at −20 °C. Frozen OCT-embedded sections were cut at 8 μm thickness and placed on microscope slides. The fluorescence of PKH26-labeled HCEC was observed under the fluorescence microscope.

## Results

### Construction of a sheet of corneal endothelial cells

**Figure 2:**
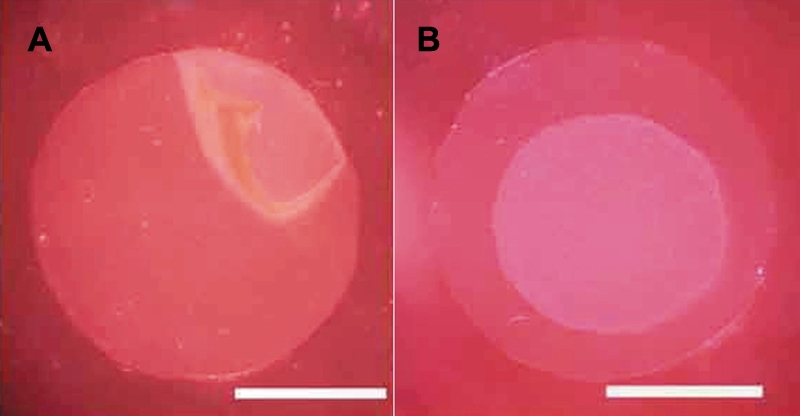
Preparation of a human corneal endothelial cell sheet. **A**: Cultured HCECs could be bluntly detached *en bloc* from the bottom of a culture insert using a spatula after EDTA treatment to the bottom side of the culture insert. **B**: The detached sheets had a circular shape with an approximately 6 mm diameter. The scale bars are equal to 5 mm.

Cultured HCECs could be bluntly detached *en bloc* from the bottom of a culture insert using a spatula after EDTA treatment of the bottom side of the culture insert. The sheets shrank after being detached from the culture insert and had a circular shape with an approximately 6 mm diameter (Figure 2).

### Histologic study

**Figure 3:**
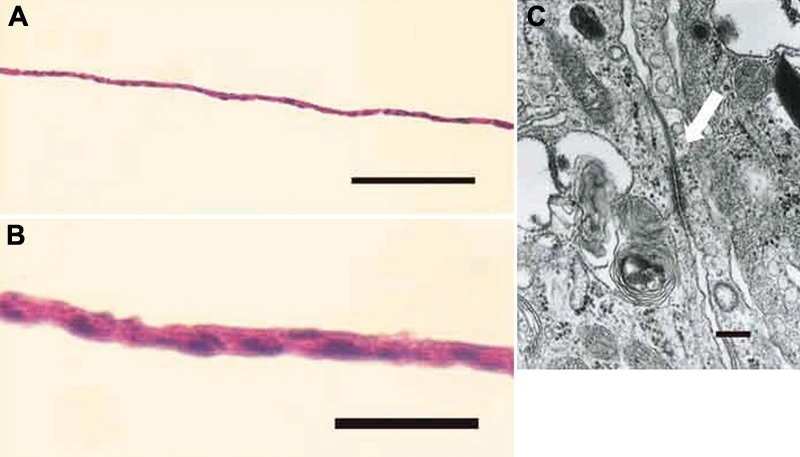
Histologic findings of the human corneal endothelial cell sheet. **A** and **B**: A cell sheet consists of a monolayer of cells that have consistent size. The scale bar in **A** is equal to 200 μm and in **B** is equal to 50 μm. **C**: Electron microscopic observation demonstrated desmosomes between cells (arrow). Bar=200 nm.

Light microscopic observation of the cell sheets with hematoxylin-eosin staining showed that the cell sheets consisted of a monolayer cells that have consistent size (Figure 3). Electron microscopic observation demonstrated desmosomes between cells (Figure 3). Those microscopic findings were similar to those in normal corneal endothelial cells.

### Immunohistochemistry

**Figure 4:**
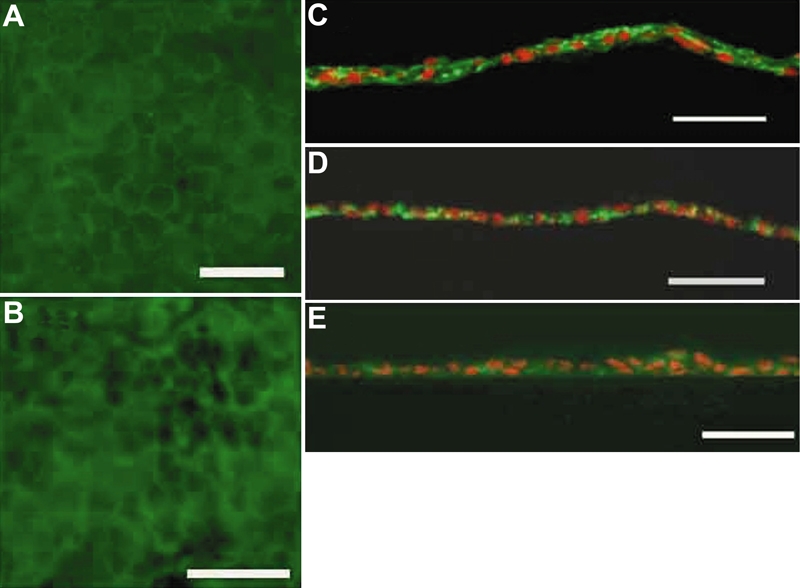
Immunohistologic findings of the human corneal endothelial cell sheet. **A** and **B**: In the flat mount immunohistochemical observation, immunostaining of both ZO-1 (**A**) and Na^+^, K^+^-ATPase (**B**) were positive at most cellular boundaries. The cellular shape demarcated with immunostaining was quasi-regular with well defined cell boundaries. **C**: In the transverse observation of extracellular matrix, immunostaining for laminin was positive at the basal side, superficial side, and intercellular space. **D** and **E**: Immunostaining of fibronectin (**D**) and type IV collagen (**E**) was weak at the surface of the sheet, but positive at the intercellular space. The scale bars are equal to 50 μm.

By flat mount immunohistochemical observation, immunostaining of both ZO-1, a tight junction-associated protein, and Na^+^, K^+^-ATPase were positive at most cell boundaries (Figure 4), suggesting proper function of cellular junctions and Na^+^, K^+^-ATPase pumps. The cellular shape demarcated with immunostaining was quasi-regular with well defined cell boundaries. The average ±standard deviation of cell density in a ZO-1 stained HCEC sheet was 2,425 \pom 83 cells/mm^2^.

In the transverse observation of extracellular matrix, immunostaining for laminin was positive at the basal side, superficial side, and intercellular space (Figure 4). Immunostaining for fibronectin and type IV collagen was weak at the surface of the sheet but positive at the intercellular space (Figure 4).

### Anterior segment observation

**Figure 5:**
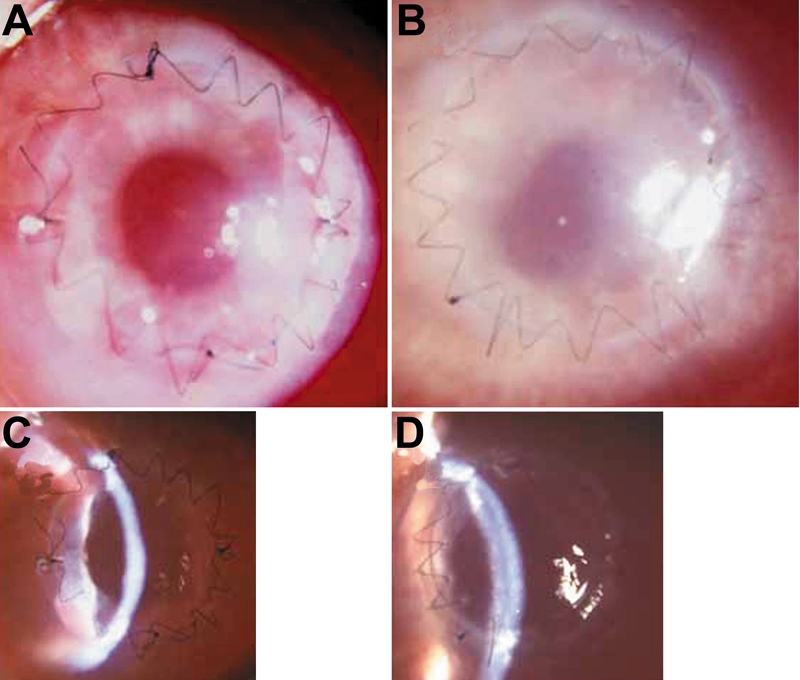
Anterior segment photographs one week after human corneal endothelial cell sheet transplantation. Representative anterior segment photographs in the HCEC sheet group (**A** and **C**) and control group (**B** and **D**) at seven days after surgery. As compared with the opaque cornea with intense stromal edema in the control group, the cornea transplanted with cultured HCEC was clear with little stromal edema.

**Figure 6:**
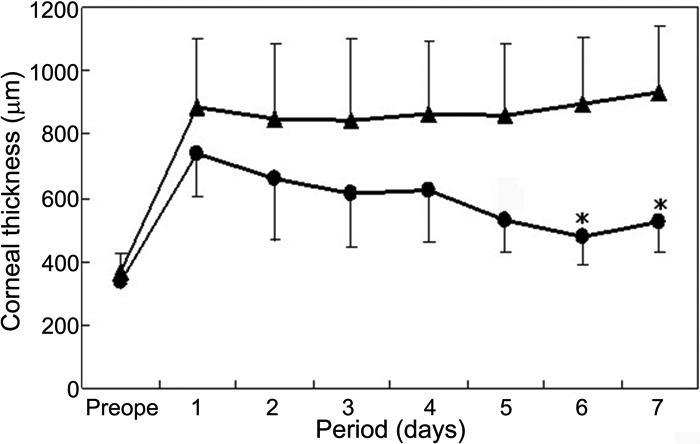
Time-course of average central corneal thickness after human corneal endothelial cell sheet transplantation. Average central corneal thicknesses in the HCEC sheet group (circle) and control group (triangle). In the control group, the mean corneal thickness remained over 800 μm throughout the one-week observation. In contrast, the mean corneal thickness rapidly decreased in the HCEC sheet group, and was significantly less than that in the control group at days 6 and 7.

After HCEC sheet transplantation, corneal edema decreased much earlier in the HCEC group than in the control group. Figure 5 shows representative anterior segment photographs in each group at seven days after surgery. As compared with the opaque corneas with intense stromal edema in the control group, the corneas transplanted with cultured HCEC were clear with little stromal edema. In the HCEC group, there was no case with primary graft failure which showed intense corneal edema at 7 days after surgery. In the control group, the mean corneal thickness remained over 800 μm throughout the one-week observation period (Figure 6). In contrast, the mean corneal thickness rapidly decreased in the HCEC sheet group and was significantly less than that in the control group at day 6 (p=0.0012) and day 7 (p=0.0016).

### Histologic examination and localization of HCEC after transplantation

**Figure 7:**
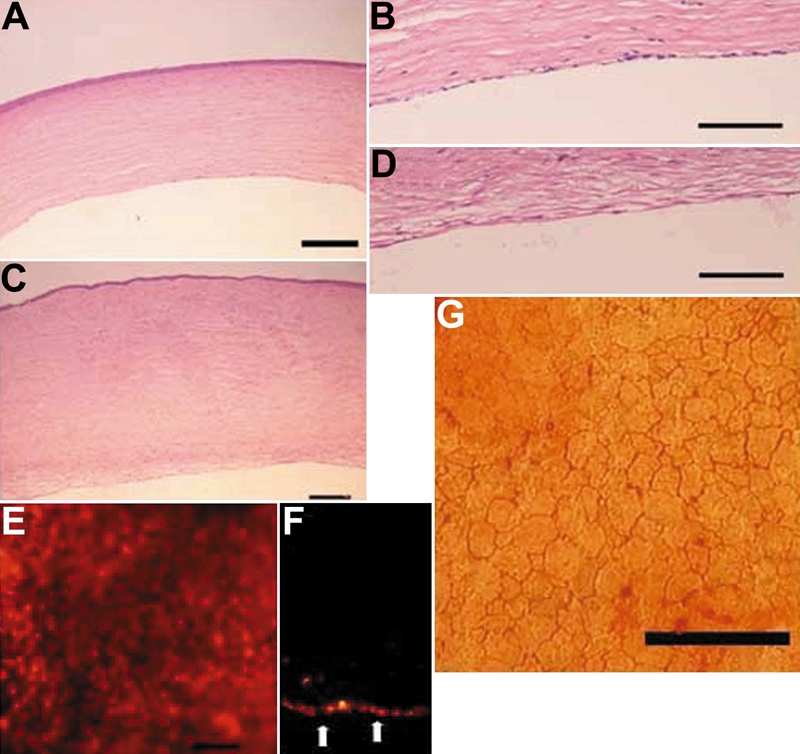
Histologic examination and localization of human corneal endothelial cells after transplantation. **A** and **B**: In the HCEC sheet group, a monolayer of continuous cells attached to the posterior surface of a rabbit cornea. **C** and **D**: On the other hand, in the control group, there was no Descemet's membrane, and endothelial cells on the posterior surface of rabbit corneas as well as the corneal stroma were thickened. **E** and **F**: Fluorescein microscopic examination of whole mount corneas (**E**) and thin sections (**F**) showed that the posterior surface of a transplanted cornea was covered with PKH26-labeled cells and apparent cell defects were not observed on the HCEC cell sheet. **G**: The average cell density in the HCEC sheet group 7 days postoperatively was 2,244 cells/mm^2^. The scale bars in **A**, **C**, and **G** are equal to 100 μm, 200 μm in **B** and **D**, and 50 μm in **E**.

In the HCEC sheet group, a monolayer of continuous cells attached to the posterior surface of the rabbit cornea and only a few cells infiltrated into the corneal stroma (Figure 7). On the other hand, there was no Descemet's membrane and endothelial cells on the posterior surfaces of the rabbit corneas in the control group, and the corneal stroma was thickened (Figure 7).

Fluorescein microscopic examination of whole mount corneas and thin sections showed that the posterior surface of the transplanted cornea was covered with PKH26-labeled cells and apparent cell defects were not observed on the HCEC cell sheet (Figure 7). The average ±standard deviation of endothelial cell density in the HCEC sheet group 7 days postoperatively was 2,244 ±21 cells/mm^2^.

## Discussion

In this study, we applied EDTA to the bottom of a cell culture insert to attenuate the attachment of HCECs and could produce a cell sheet of HCEC by bluntly detaching the cell sheet with a spatula after the application of EDTA. There have been reports of several kinds of methods to produce cell sheets, such as the use of proteolytic enzymes [30,31], biodegradable scaffolds [32,33], and temperature-sensitive culture dishes [34,35]. Importantly, our technique employs EDTA which is widely used in cell culture and is inexpensive. Moreover, because EDTA has been clinically used in chelation therapy for cardiovascular diseases [36-38] and calcific band keratopathy [39], it may be considered for clinical use. However, the safety of EDTA for intraocular use in the amounts used in this study has not been established. Thus the relative advantages or disadvantages of this method compared to other methods of creating a cell sheet are unknown at this time.

Although we cultured HCEC for a relatively short period (one week), their morphologic properties were similar to HCEC in vivo. The HCEC sheet consists of a monolayer sheet and has numerous desmosomes. The average cellular density one week after transplantation was 2,244 cells/mm^2^, which is slightly lower than that of normal HCECs in vivo, but is enough to maintain corneal clarity in clinical situations. Immunohistochemistry demonstrated ZO-1 and Na^+^, K^+^-ATPase at the cell boundaries, suggesting proper intercellular junctions and cellular pump function. Moreover, extracellular matrix proteins, such as fibronectin, laminin, and type IV collagen, which are present around HCEC in vivo, were demonstrated via immunohistochemistry in the cell sheet after EDTA treatment. These findings were similar to those in HCECs in vivo and cultured HCECs [22,40-42], indicating that EDTA treatment has the advantage of preserving the conditions and environment of HCECs when compared to enzymatic treatment.

The function of the HCEC sheet was examined in vivo. Corneal transparency was recovered and the average corneal thickness was 526 μm at one week after surgery of eyes in which a HCEC sheet was transplanted, whereas the corneal transplant was edematous and the average corneal thickness was 930 μm at one week in the control group. The histologic examination after the transplantation showed that the posterior surface of the corneal graft was covered with endothelial cells, and cellular infiltration to the posterior portion of corneal stroma was greatly reduced in the HCEC sheet transplantation group relative to the control group. These results showed that the HCEC sheet functions well in vivo and contributes to the maintenance of corneal clarity.

Because the HCEC sheet was produced by bluntly detaching cells, the sheet might experience mechanical damage. The flat mount observation, however, did not show any acellular areas in the sheet and immunohistochemical analysis showed that the ECM remained around the HCECs. Additionally, the average cellular density one week after the transplantation was 2,244/mm^2^, which seems satisfactory considering that surgical manipulations might affect cells on the sheet. These results suggest that the detachment maneuver of the HCEC sheet caused little damage to the cells.

In this study, we examined the in vivo function of the HCEC sheet for one week after the transplantation. Although a longer-term observation period would be desirable, such prolongation of the observation period might potentially be associated with confounding effects such as xenograft-related immune response and proliferation and migration of native host corneal endothelial cells surrounding the graft. During the one week observation period, however, we observed significant differences in corneal clarity and corneal thickness between the eyes that received HCEC sheets and the controls, suggesting that a one-week follow-up is enough to confirm the in vivo functionality of the HCEC sheet.

In this study, the surgical procedure was similar to penetrating keratoplasty because the fragility of the HCEC sheets limits the choice of surgical procedures. However, if we have to perform surgery similar to penetrating keratoplasty when we use the cell sheet, the benefit of using the cultured HCEC sheet is decreased. To enhance the benefit of using the cultured HCEC sheet, we might need to develop a new surgical procedure by combining the cultured HCEC sheet and surgical procedures such as Descemet's stripping and endothelial keratoplasty and deep lamellar endothelial keratoplasty.

In conclusion, we produced HCEC sheets by applying EDTA to the bottom side of the culture insert and confirmed in vitro and in vivo functionality of the HCEC sheet. This novel technique for producing a HCEC sheet might be useful in the field of regenerative medicine for the reconstruction of corneal endothelium.
